# Effectiveness of an “Exclusive Intervention Strategy” to increase medical male circumcision uptake among men aged 25–49 years in South Africa

**DOI:** 10.1186/s12889-018-5729-6

**Published:** 2018-07-13

**Authors:** Jonathan M. Grund, Candice M. Chetty-Makkan, Sibuse Ginindza, Reuben Munyai, Helen Kisbey-Green, Mpho Maraisane, Salome Charalambous

**Affiliations:** 1 Centers for Disease Control and Prevention, 1600 Clifton Road NE, MS-E04, Atlanta, GA 30333 USA; 2 The Aurum Institute, Johannesburg, Gauteng Province South Africa; 3 The School of Public Health, University of the Witwatersrand, Johannesburg, South Africa

**Keywords:** Male circumcision, Demand creation, South Africa, HIV prevention

## Abstract

**Background:**

South Africa introduced medical male circumcision (MMC) to reduce HIV incidence. Mathematical modeling suggested that targeting MMC services to men aged 20–34 years could provide the most immediate impact on HIV incidence. However the majority of MMCs performed have been among males aged ≤25 years. We evaluated an intervention package to increase MMC uptake among men aged 25–49 years.

**Methods:**

We conducted a pre-post study to compare the proportion of men (aged 25–49 years) presenting for MMC during the formative (Phase 1) and intervention (Phase 2) phases in Ekurhuleni, Johannesburg, South Africa. The intervention included infrastructure changes that separated adults from adolescents at the MMC site, an exclusive men’s health club, adult-specific demand generation materials, and discussions with community members.

**Results:**

Overall 2817 enrolled in the study with 1601 from Phase 1 and 1216 in Phase 2. A higher proportion of participants aged 25–49 years accessed MMC in Phase 2 compared to Phase 1 (59.4% vs. 54.9%; Prevalence Ratio = 1.08; 95% Confidence Interval: 1.01–1.15; *p* = 0.019). Participants with multiple partners in the past 12 months in Phase 2 were more likely to access MMC services compared to participants in Phase 1 (unadjusted Odds Ratio, 1.37; 95% CI:1.17–1.61; *p* < 0.001). After adjusting for age, multiple partners remained a risk factor in Phase 2 (adjusted OR, 1.39; 95% CI: 1.18–1.63; *p* < 0.001).

**Conclusions:**

The “Exclusive Intervention Strategy” was associated with a slight increase in the proportion of participants aged 25–49 years accessing MMC services, and an increase in those with HIV risk behaviors, during the intervention phase. These findings may provide important insights to overcoming barriers for accessing MMC services among men aged 25–49 years.

**Trial registration:**

The study is registered at ClinicalTrials.gov, number NCT02352961.

## Background

Three randomized controlled trials confirmed that medical male circumcision (MMC) prevents HIV infection in men through heterosexual sex by at least 60% [1–4]. In 2007, the World Health Organization (WHO) and the Joint United Nations Programme on HIV/AIDS (UNAIDS) recommended male circumcision as a compelling HIV prevention intervention. Their recommendations specified that MMC should be offered in settings with low male circumcision coverage and high HIV prevalence [5]. Fourteen countries were determined to be prioritized for rapid MMC scale-up. It was estimated that providing 20.3 million MMCs among HIV-negative males aged 15–49 years in the fourteen priority countries could avert 3.36 million new HIV infections and result in $16.5 million in averted HIV treatment costs [6]. Working with Ministries of Health and MMC service delivery implementing partners, WHO estimates that nearly 12 million MMCs have been conducted through 2015, and 61% of those are among males aged ≥15 years [7].

South Africa is a priority country for MMC scale-up, as it has a national adult HIV prevalence of 17.9% [8] and has conducted approximately 1.86 million MMCs, which is 43% of their target of 4.3 million [7]. While the prevalence of male circumcision is estimated to be 54.0% nationally, there is high level of provincial variability [7]. The majority of MMCs performed among 15–49 year olds from 2010 to 2014 were in the provinces of KwaZulu-Natal, Gauteng, and Mpumalanga totaling more than 1.2 million [9]. WHO recommends MMC for males aged 15–49 years in the priority countries, but 65% of all MMCs supported by PEPFAR from 2010 to 2012 were among males aged 15–19 years. Similarly, South Africa has conducted almost 2 million MMCs for HIV prevention since 2009, though uptake has overwhelmingly attracted younger clients [10, 11].

Overall HIV prevalence in South Africa increases significantly with age: 0.7% of males aged 15–19 years are HIV positive, and prevalence increases to 17.3% and 25.6 for males aged 25–29 and 30–34 years, respectively [11]. Studies have indicated that HIV risk behaviors also increase among older men in South Africa [11]. A total of 21.1% of black African males aged 25–49 years reported that they had more than one sexual partner in the past 12 months, compared with the national average of 12.6% among all races [11]. Condom use was also significantly lower among men aged ≥25 years compared with younger males [11]. Modeling exercises have determined that in order for MMC to confer the most cost-effective and immediate impact on HIV incidence in South Africa, MMC for males aged 20–34 years must be prioritized and demand creation activities should specifically target these uncircumcised men, as MMC uptake remains low among this group [9].

Client-level barriers to MMC uptake among males aged ≥18 years are well documented in qualitative research in several countries implementing MMC for HIV prevention. The most common barriers among men aged ≥20 years include: shame at having to share waiting areas with younger MMC clients [12], confusion of MMC and traditional circumcision [13], concern that MMC is against cultural and ethnic identities [14], fear of pain and potential complications [13–15], difficulty in adhering to sexual abstinence for 6 weeks following MMC [13], and loss of income during the healing period [15, 16]. Given the young age profile of MMC clients in South Africa, it is critical that demand creation activities address these client- and facility-level barriers in order to attract older men and maximize MMC’s impact on HIV incidence.

This study reports the results from a novel recruitment strategy to increase MMC uptake among men aged 25–49 years at an MMC facility in Ekurhuleni, Gauteng Province, a district near Johannesburg, South Africa. A pre-post study design was conducted from April 2014–November 2015 at the Aurum Institute Winnie Mandela Male Sexual Health Clinic. This study assessed whether a targeted approach to demand creation could increase MMC uptake among men aged 25–49 years with HIV risk behavior. The study’s aim was to evaluate the effectiveness of an “Exclusive Intervention Strategy”, comprised of customized MMC service delivery, to increase MMC uptake in men aged 25–49 years.

## Methods

The study was conducted at a fixed clinic in a peri-urban area, where male health services including MMC are provided routinely. This implementation science study was embedded within the routine MMC service delivery program that included HIV testing services (HTS), registration for MMC, physical and genital examination, group counselling, one-on-one counselling (where separate informed consents were administered for the MMC procedure and for data to be used for research purposes), the MMC surgical procedure, post-operative observations, and discharge.

We obtained approval for the study from the Witwatersrand Human Research Ethics Committee, University of Witwatersrand (Approval Number: M130711) and the research committee of the Centers for Disease Control and Prevention (protocol number 6546). The study was registered on clinicaltrials.gov (NCT02352961). Voluntary written informed consent was obtained from all participants, and, in the case of illiterate participants, a thumbprint to acknowledge understanding in the presence of a witness was obtained.

The study consisted of two phases where Phase 1 and 2 took place in (1 April - 30 September 2014) and (22 June - 30 November 2015) respectively. For Phase 1, quantitative data was collected to determine the baseline estimate of risk factors for men (aged 18–49 years) who accessed routine MMC services. There was also a qualitative component where the barriers and motivators of MMC from male and female perspectives were explored to inform the intervention. Outcomes from the quantitative phase are reported here while the qualitative results are published elsewhere [17]. Interventions for Phase 2 were developed using the themes from the qualitative phase. Based on the qualitative findings and programmatic experiences, the intervention package included infrastructure changes at the MMC site that separated adults (aged ≥18 years) from adolescents (10–17 years old), an exclusive men’s health club (lounge area, free Wi-Fi access, VIP Facebook page by invite only, and shoe shine services) that was only available at the study clinic, adult-specific community demand generation material (billboards, pamphlets, posters and branded condoms) and “Imbizo” discussions with community members. “Imbizo” refers to traditional forums where elders discuss important issues with the community, and we used this concept to arrange broader discussions with men and women about MMC. The “Exclusive Intervention Strategy” was implemented only during Phase 2. The primary aim of Phase 2 was to determine if the “Exclusive Intervention Strategy” would increase the proportion of men (25–49 years) who accessed MMC services when compared to Phase 1.

**Fig. 1:**
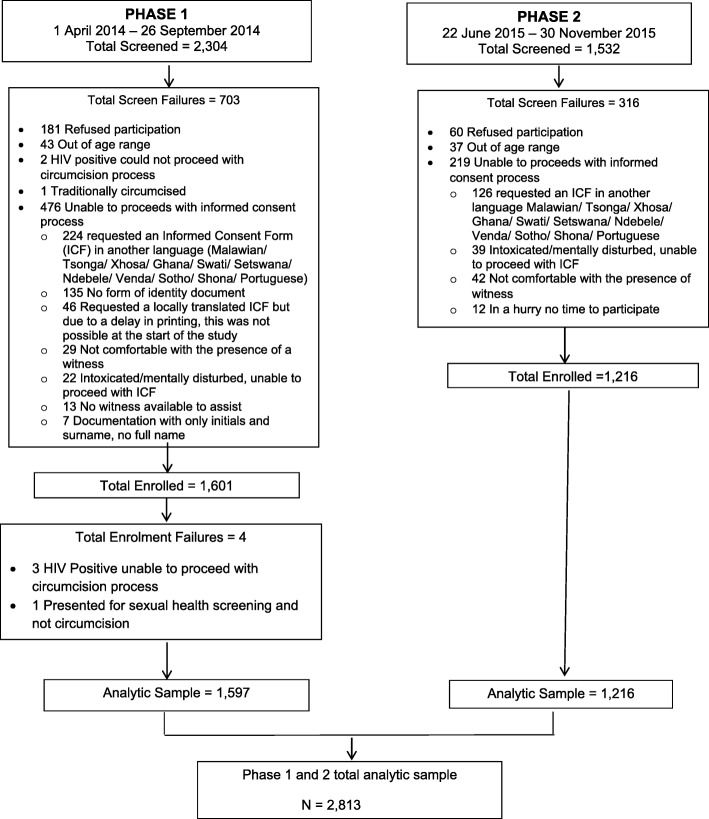
Flow of enrolment diagram for Phase 1 and Phase 2 of the Imbizo study

Study recruitment for both phases targeted men (aged ≥18 years) who were interested in MMC. Outreach teams distributed posters, pamphlets, branded condoms; held campaigns with the community; and used media such as advertising on taxis and trains to invite men to be circumcised. Men interested in MMC were given a clinic appointment. After registration, potential study participants were screened for study eligibility. Screening criteria included men who were interested in being circumcised, aged 18–49 years old, and able to communicate in English, IsiZulu, or Sepedi. Participants were given information about the study, and eligible men who were interested in participating provided written informed consent (Fig. 1). Demographic information (e.g. employment, financial and relationship status), sexual partner history, condom use, and the primary reasons for pursuing MMC from a set of multiple options were subsequently collected.

### Statistical analysis

The statistical analysis was conducted using STATA (version 14, StataCorp LP, College Station, Texas). For the study data, the questionnaire extracts from Phase 1 and 2 were merged with the medical information that was recorded during the routine MMC procedures for the study sample. From the study data, we compared the proportion of men aged 25–49 years to all enrolled participants (18–49 years) between Phase 1 and 2. Socio-demographic and sexual risk characteristics of men (18–49 years) at enrollment were described for Phase 1 and 2. A second data extract included all men who went through the routine MMC procedures (irrespective of study enrollment) and who consented for their routine data to be used for research. We analyzed the routine data in order to obtain the proportion of all men (aged 25–49 years) who accessed MMC services over the period overlapping with the study phases. For categorical variables (e.g. methods of recruitment), the frequencies and percentages are presented while the median and inter-quartile ranges are presented for continuous variables (e.g. participant age). The effect of study phase on the proportion of men (25–49 years) presenting for MMC was calculated using Fisher’s Exact test and the prevalence ratio with a 95% confidence interval (CI). Univariate logistic regression, adjusting for age, was used to investigate the association of phase on sexual risk behaviors. Having more than one sexual partner in the last 12 months, partner greater than 5 years younger, no condom use during the last sex act, and having a sexually transmitted infection (STI) were the risk factors identified prior to analysis.

### Sample size

It was assumed that approximately 40–55% of men (≥18 years) who come to the facility for MMC would be within the target age range of 25–49 years with a variance of 2.5%. The minimum number of men to be recruited was 1537 for a minimum power of 80% at 5% significance level.

## Results

A total of 3836 men aged 18–49 years were screened: 2304 in Phase 1 and 1532 in Phase 2. Of those screened, a total of 2817 (73.4%) enrolled in the study with 1601 in Phase 1 and 1216 in Phase 2. The enrolment to screening ratios for Phase 1 and Phase 2 were 69.5 and 79.4% respectively. The primary reason for screen failures in both phases was the inability to communicate in one of the study languages (*n* = 350). Of those enrolled, 2813 (99.9%) were included in the analysis of the primary outcome with 1597 (56.8%) from Phase 1 and 1216 (43.2%) from Phase 2 (Fig. 1).

In Phase 1, the median age was 25 years, 28.7% had more than one sexual partner, 24.6% had a sexual partner > 5 years younger, 47.8% did not use a condom at last sex, and 2.8% were treated for an STI in the past 6 months. In addition, 44.6% were employed and 61.5% considered themselves as financially secure as their peers. In Phase 2, the median age was 26 years, 35.6% had more than one sexual partner, 27.2% had a sexual partner > 5 years younger, 49.8% did not use a condom at last sex, and 2.9% were treated for an STI in the past 6 months. Additionally, 46.1% were employed and 64.8% considered themselves as financially secure as their peers (Table 1).

**Table 1:** Participant demographic characteristics and sexual risk behaviours from Phase 1 and 2 of the Imbizo study

Variable	Categories	Phase 1 *N* = 1597 n (%)	Phase 2 *N* = 1216 n (%)
DEMOGRAPHICS			
Age (in years)	Median (IQR)	25 (21–30)	26 (21–32)
Employment Status	Unemployed	484 (30.3)	291 (23.9)
	Employed	712 (44.6)	560 (46.1)
	Student	282 (17.7)	257 (21.1)
	Self-Employed	96 (6.0)	91 (7.5)
	Other	12 (0.8)	16 (1.3)
	Missing	11 (0.7)	1 (0.08)
Family Finances	Not enough food/clothes	158 (9.9)	149 (12.3)
	Enough food/clothes but short on other	689 (43.1)	474 (39.0)
	Basics but not enough for expensive things	702 (44.0)	541 (44.5)
	Enough to save or buy expensive things	47 (2.9)	48 (3.9)
	Missing	1 (0.06)	4 (0.3)
Peer Financial Comparison	Poorer than others	389 (24.4)	262 (21.5)
	About the same as others	982 (61.5)	788 (64.8)
	Better off than others	217 (13.6)	160 (13.2)
	Missing	9 (0.6)	6 (0.5)
RELATIONSHIP HISTORY AND SEXUAL RISK BEHAVIOUR			
Relationship Status in the last 12 months	No partner or one steady/casual partner at same given time	1136 (71.1)	781 (64.2)
	> 1 partner at same given time	459 (28.7)	433 (35.6)
	Missing	2 (0.1)	2 (0.2)
Live-in partners in the last 12 months	No	917 (57.4)	695 (57.2)
	Yes	538 (33.7)	433 (35.6)
	Not applicable-no partners in the last year	141 (8.8)	86 (7.1)
	Missing	1 (0.06)	2 (0.2)
How many partners did you have in the last 6 months?	None	216 (13.5)	182 (15.0)
	One	874 (54.7)	573 (47.1)
	Two	285 (17.8)	233 (19.2)
	More than two	189 (11.8)	124 (10.2)
	Missing	33 (2.1)	104 (8.6)
In the last 6 months how often have you used a condom?	Never	504 (31.6)	328 (27.0)
	Sometimes	552 (34.6)	371 (30.5)
	Most times	84 (5.3)	86 (7.1)
	Every time	376 (23.5)	249 (20.5)
	Missing	81 (5.1)	182 (15.0)
How old was your last sexual partner?	About same age or > 5 years older	1159 (72.6)	824 (67.8)
	> 5 years younger	393 (24.6)	331 (27.2)
	Not applicable--never had sex	40 (2.5)	56 (4.6)
	Missing	5 (0.3)	5 (0.4)
Did you use a condom the last time you had sex?	No	763 (47.8)	606 (49.8)
	Yes	789 (49.4)	550 (45.2)
	Not applicable-never had sex	42 (2.6)	56 (4.6)
	Missing	3 (0.2)	4 (0.3)
Suspect last partner of infidelity	No	773 (48.4)	513 (42.2)
	Yes	368 (23.0)	280 (23.0)
	Not sure	409 (25.6)	364 (29.9)
	Not applicable-never had sex	40 (2.5)	56 (4.6)
	Missing	7 (0.4)	3 (0.2)
SUBSTANCE USE HISTORY			
Spent time in a tavern in the last 12 months	Never	751 (47.0)	557 (45.8)
	Occasionally	606 (37.9)	486 (40.0)
	Frequently	240 (15.0)	170 (14.0)
	Missing	0 (0)	3 (0.2)
Drank alcohol in the last 12 months	Never	586 (36.7)	401 (33.0)
	Occasionally	739 (46.3)	597 (49.1)
	Frequently	272 (17.0)	216 (17.8)
	Missing	0 (0)	2 (0.2)
SEXUALLY TRANSMITTED INFECTIONS			
Have you been treated for STIs past 6 months	No	1531 (95.9)	1092 (89.8)
	Yes	45 (2.8)	35 (2.9)
	Missing	21 (1.3)	89 (7.3)
Current treatable STI as diagnosed by clinical staff member	No	1538 (96.3)	1099 (90.4)
	Yes	30 (1.9)	20 (1.6)
	Missing	29 (1.8)	97 (8.0)

From the study data, a higher proportion participants aged 25–49 years accessed MMC services in Phase 2 when compared to Phase 1 (59.4% vs. 54.9%; PR = 1.08; 95% CI: 1.01–1.15; *p* = 0.019, Table 2). The proportion of men who were circumcised from the routinely collected data also showed that a higher proportion 25–49 year olds accessed MMC services (56.8% vs. 53.2%; PR 1.07; 95% CI: 1.04–1.10; *p* < 0.001). When comparisons were done to assess MMC uptake during overlapping months of Phase 1 and 2 (23 June – 30 September), the proportion of participants aged 25–49 years were greater in Phase 2 vs. Phase 1 (57.8% vs. 52.8%; PR = 1.10; 95% CI: 1.01–1.19; *p* = 0.032) (data not shown). Although there was an overall increase in the proportion of men (25–49 years) who accessed MMC services in Phase 2, the proportion of the increase was not significant. In addition, more men aged 25–49 years were circumcised per day during Phase 1 compared to Phase 2 (Table 2). Phase 1 comprised of 182 days (April 1, 2014 – September 30, 2014), and 4874 men were circumcised during this period. Phase 2 comprised of 161 days (June 22, 2015 – November 30, 2015), and 2750 men received MMC. The crude rates per calendar day is 26.8 men in Phase 1 and 17.1 men in Phase 2.

**Table 2:** Proportion and number of circumcised clients in different age-groups, by study dates and routine MMC services of the Imbizo study

IMBIZO RESEARCH STUDY					
Study Phase (recruitment dates)		18–24 years	25–49 years	Prevalence Ratio comparing Phase 2 to Phase 1 (25–49 years)	
				PR (95% CI)	*p*-value
Phase 1 (01 Apr 2014–30 Sep 2014) N = 1597	n (%)	720 (45.1)	877 (54.9)	1	0.019
Phase 2 (22 Jun −30 Nov 2015) N = 1216	n (%)	494 (40.6)	722 (59.4)	1.08 (1.01–1.15)	
MMC ROUTINE DATA					
Routine data (dates coinciding with Phase recruitment)		18–24 years	25–49 years	Prevalence Ratio comparing Phase 2 to Phase 1 (25–49 years)	
Phase 1 (01 Apr 2014–30 Sep 2014) *N* = 9169	n (%)	4295 (46.8)	4874 (53.2)	1	< 0.001
Phase 2 (22 Jun 2015–30 Nov 2015) *N* = 4839	n (%)	2089 (43.2)	2750 (56.8)	1.07 (1.04–1.10)	

**Fig. 2:**
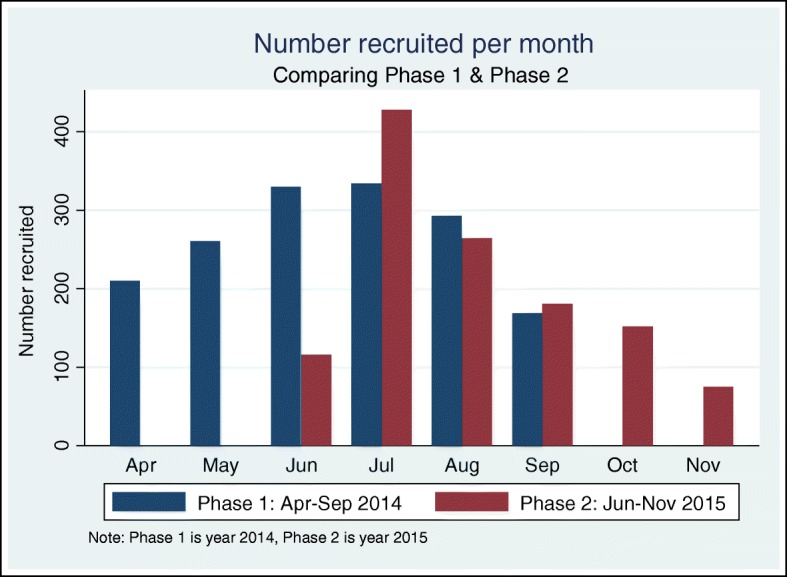
Number of MMC clients recruited per month for Phase 1 (April–September 2014) & Phase 2 (June–November 2015)

Figure 2 shows the monthly results of participants presenting for MMC. In both phases, the highest number of participants presented during July (762) and August (557). During these months in Phase 1, the majority of participants were aged 25–49 years, whereas younger clients were more common during the other months. For Phase 2, the age distribution for participants presenting for MMC is similar to Phase 1 but more pronounced in August and September, as 68.6 and 73.5% of clients were aged 25–49 years respectively.

The association of study phase on sexual risk behaviors showed that participants from Phase 2 with multiple partners during the same or at different times over the past 12 months were more likely to access MMC services when compared to Phase 1 (unadjusted OR, 1.37; 95% CI:1.17–1.61; *p* < 0.001). After adjusting for age, multiple partners remained as a risk factor in Phase 2 (adjusted OR, 1.39; 95% CI: 1.18–1.63; *p* < 0.001). The other HIV risk behaviors (having a sexual partner who was greater than 5 years younger, not using a condom use at last sex, and reported STIs in the past 6 months) were not associated with the study phases (Table 3).

**Table 3:** Logistic regression investigating the association of sexual risk behaviours for Phase 1 and Phase 2

	Relationship Status in the last 12 months^a^		Age of last sexual partner^b^		Used a condom the last sex act^c^		Report of any STIs in the past 6 months^d^	
	No partner or one steady/casual partner at any given time	> One partner at some given time (1)	About same or > 5 years older	> 5 years younger (1)	Yes	No (1)	Had an STI in the past 6 months	No STI in the past 6 months (1)
Phase 1 (23 Jun −30 Sep 2014) *N* = 1597	1136 (71.1)	459 (28.7)	1159 (72.6)	393 (24.6)	789 (49.4)	763 (47.8)	1531 (95.9)	45 (2.8)
Phase 2 (22 Jun −30 Sep 2015) N = 1216	781 (64.2)	433 (35.6)	824 (67.8)	331 (27.2)	550 (45.2)	606 (49.8)	1092 (89.8)	35 (2.9)
Unadjusted OR (95% CI) Phase 2 to Phase 1, *p*-value	1.37 (1.17;1.61), *p* < 0.001		1.18 (1.00;1.41), *p* = 0.05		1.14 (0.98;1.33), *p* = 0.09		1.09 (0.70;1.71), *p* = 0.71	
Adjusted for age^e^ OR (95% CI) Phase 2 to Phase 1, *p*-value	1.39 (1.18;1.63), *p* < 0.001		1.02 (0.85;1.24), *p* = 0.81		1.06 (0.91;1.25), *p* = 0.42		1.09 (0.69;1.71), *p* = 0.71	

^a^two missing relationship status in each phase

^b^42 not sexually active in the past 12 months in Phase 1, 56 not sexually active in the past 12 and 5 missing in Phase 2

^c^42 not sexually active in the past 12 months, 2 did not know partner’s age and 1 missing in Phase 1. 56 not sexually active in the past 12 months in Phase 2, 2 did not know, I, 4 missing in Phase 2

^d^110 excluded in the analysis because of missing data (21 Phase 1, 89 Phase 2)

^e^Adjusted for age in four categories; 18–20 years, 21–24 years, 25–30 years, 31–49 years

Participants from both study phases also reported primary reasons for attending the MMC clinic. Media, which included Facebook, billboards, flyers, and all other promotional materials that were part of the intervention, was the highest ranked reason at 20.3%. Hygiene was the second highest ranked reason at 17.9%, followed by recommendations from other clients or relatives who underwent MMC (17.1 and 13.6% respectively), and recommendations from partners (11.0%).

## Discussion

Our study showed that an “Exclusive Intervention Strategy”, which included infrastructure improvements, outreach branded materials, access to a VIP Facebook page, invitations to community discussion groups, and special services in an exclusive men’s lounge, slightly increased the proportion of participants aged 25–49 years who accessed MMC services. The “Exclusive Intervention Strategy” may provide important insights to overcoming barriers for accessing MMC services among men aged 25–49 years and creates an opportunity to identify men at high risk of HIV who could be targeted for future HIV prevention interventions.

Participants in the intervention phase were older (median age 26 vs. 25 years), more likely to be employed (46.1 vs 44.6%), and more financially secure (64.8% vs. 61.5%). In addition, men with greater risk behaviors were more likely to come for MMC in Phase 2 over Phase 1: participants with multiple concurrent partners (35.6% vs. 28.7%), no condom use at last sex (49.8% vs. 47.8%), and overall time spent in a tavern (53.8% vs. 52.9%).

However, routinely collected site-level data suggest that greater proportions of 25–49 year olds were coming to the MMC site (56.8% vs. 53.2%), though to a lesser extent than among study participants. The increases in men aged 25–49 years coming to the MMC site could be attributed to the targeted messaging that was designed to appeal to men aged 25–49 years during the intervention phase, but it could also be that some clients aged 18–25 years were attracted to the targeted messages. It is also possible that more “older” men were interested in pursuing MMC during the winter months, as seasonality is a strong predictor of MMC uptake in South Africa. July and August include school holidays, which could suggest that the separation of clients by age is a facilitator for men aged ≥25 years. Also, it is possible that the lower crude rate of men aged 25–49 getting MMC per day is lower in Phase 2 vs. Phase 1 is due to increasing saturation of MMC in the communities close to the study site in Ekurhuleni.

Our study adds to growing research from sub-Saharan Africa about MMC and demand creation to encourage hard-to-reach men to pursue MMC. Thirumurthy et al. (2014) offered economic incentives to men aged 25–49 years in Nyanza region, Kenya on the condition that they were circumcised within 2 months at one of the nine MMC study sites. Study groups included participants who received food vouchers worth ~$2.50, ~$8.75, and ~$15.00 and no compensation (control). MMC uptake was strongest in the groups that received ~$8.75 and ~$15.00 (AOR 4.3; 95% CI, 1.7–10.7 and AOR 6.2; 95% CI, 2.6–15.0) [18]. In Tanzania, Wambura et al. targeted demand creation messaging, MMC site-level alterations, and engagement of female sexual partners was investigated to increase uptake in men 20–34 years. The study had a strong effect in Tabora region (Risk Ratio = 2.39, 95% CI 1.7–3.4) but no significant effect in Njombe region (RR = 0.77; 95%CI 0.4–1.6) [19]. In South Africa, Wilson et al. conducted a randomized controlled trial with men aged ≥18 years in Soweto. The intervention arms included participants receiving ~$10.00, additional information about MMC, or a “challenge” question to encourage participants to come for MMC. The compensation and “challenge” question arms resulted in significantly higher MMC uptake [OR 5.30 (95% CI, 2.20–12.76) and 2.70 (CI, 1.05–6.91, respectively [20]. Also in South Africa, Auvert et al. offered uncircumcised men home-based motivational interviews that addressed common barriers to MMC [21]. For men opting for circumcision, they received financial compensation for lost work time. This study resulted in a relative increase of MMC prevalence of 43.6%.

Our findings are aligned with other MMC demand creation studies to increase MMC uptake in sub-Saharan Africa. The studies with the largest effect sizes are those that offered financial compensation, which suggests that the costs associated with travel to the MMC clinic and time taken off from work during recovery from MMC surgery might be substantial barriers preventing some men from pursuing MMC [18, 20]. However offering financial compensation along with MMC is challenging to implement on a large scale, and the additional program costs may result in higher unit costs. The Tanzanian study’s overall results were more modest and similar to ours, which may suggest that additional research should include enhanced demand creation, clinic modifications, and some financial incentives to appeal to potential clients with varied barriers [19].

Our study had several strengths and limitations. The strength of our study is that the intervention package was informed by qualitative research that incorporated user preferences with common cultural practices to appeal to the community. The intervention package addressed barriers that prevented “older” men from coming for MMC, including queuing with adolescent clients, and it appealed to men’s interest in having an “exclusive” experience at a men’s health clinic, including shoe shine services, lounge access, etc. The study’s limitations included the study design that involved different populations in Phases 1 and 2 who could have had different reasons for coming for MMC. The study site was also a large, peri-urban men’s health clinic, so generalizing these results might be challenging for rural health facilities that might experience challenges separating services or establishing an exclusive lounge area. In addition, MMC is a well-known health intervention among males in Gauteng Province, South Africa, so the intervention may need to be altered depending on MMC coverage in another population. Finally, the study instruments did not allow for a factor analysis to attribute specific components of the intervention that were most responsible for the increase in MMC uptake among men aged 25–49 years.

## Conclusion

Our study showed that a targeted demand creation strategy that separated adolescent and adult men and offered “exclusive” services to men aged 25–49 years slightly increased the proportion of clients aged 25–49 years getting MMC at a study facility in South Africa. The components of this intervention package should inform novel demand creation strategies to recruit older and higher risk men to MMC for HIV prevention services. Future demand creation studies should assess and incorporate client-level preferences to increase impact.

## Abbreviations

CI: Confidence Interval
HIV: Human Immunodeficiency Virus
HTS: HIV Testing Services
IQR: Interquartile Range
MMC: Medical Male Circumcision
OR: Odds Ratio
PR: Prevalence Ratio
RR: Risk Ratio
STI: Sexually Transmitted Infection
UNAIDS: Joint United Nations Programme on HIV/AIDS
VIP: Very Important Person
WHO: World Health Organization
